# Research, Teaching, and Knowledge Transfer Assessment in Spain: Strategies and Results

**DOI:** 10.3389/frma.2022.817031

**Published:** 2022-06-29

**Authors:** Domingo Docampo, Vicente Safón, Carlos Albert

**Affiliations:** ^1^AtlanTTic Research Center for Telecommunication Technologies, University of Vigo, Vigo, Spain; ^2^Department of Management, University of Valencia, Valencia, Spain; ^3^IVIE, Valencia, Spain; ^4^Department of Economic Analysis, University of Valencia, Valencia, Spain

**Keywords:** research assessment and evaluation, teaching assessment, research policy and governance, Spain, university, higher education system (HES)

## Abstract

In Spain, faculty members' and researchers' activities are assessed for three main reasons: applying for university teacher and researcher positions, developing their academic careers, and calculating their individual remunerations. This assessment covers three dimensions of university activity: teaching, research, and knowledge transfer. Over the last 20 years, the level of career-related requirements has been raised considerably with the introduction of a habilitation system in 2001, which was followed by a new accreditation system in 2007, both national in scope and beyond the control of individual universities. On the other hand, during the last three decades, three distinct financial incentives have been introduced for tenure-track positions: one for teaching achievements, which was introduced in 1989, is awarded every five years, and is managed by the universities; a research incentive that was created in 1989, is available every 6 years, and is managed outside the universities; and another one that has been recently created to reward knowledge-transfer achievements. In this article, we analyze the results achieved through these instruments and conclude that the incentive schemes taken out of the academic control of university governance were the most successful ones.

## Introduction

Over the last 30 years, the Spanish university and research systems have experienced important reforms, with excellent results, especially in published research. In this paper, we analyze these reforms and make a critical judgment about the results. We also highlight the instruments that have had the greatest influence on individual behavior, specifically, on people's careers and salaries.

The primary source of aggregated university data used in this study is the Integrated University Information System (SIIU in Spanish), a platform built over the last decade for collecting, processing, analyzing, and disseminating data. This platform began to be developed in 2010 as a result of the adaptation of the Spanish university system to the framework established by the European Higher Education Area (EHEA). It includes data from the Spanish autonomous communities, universities, and the national government. Furthermore, it is an essential tool for obtaining the official university statistics implemented in the Spanish National Statistical Plan and is the only platform that provides homogeneous and comparable information^1^.

In the following sections, we review the main instruments that were introduced to improve research (Section Research), teaching (Section Teaching), and knowledge transfer practices (Section Knowledge Transfer). The final section (Section Discussion and Conclusions) is devoted to summarizing our conclusions.

## Research

Research is assessed both in terms of people's access to scientific and university careers and, above all, in terms of the results achieved throughout the career itself. Within the latter realm, the assessment considers two dimensions: the evaluation prior to research and teaching contracts or positions awarded and the evaluation of research performance to pay researchers an individual productivity bonus. Given that a 6-year research period is taken into account, this incentive is referred to as *research sexennial*.

### Evaluation for Accessing Research Positions

**Figure 1:**
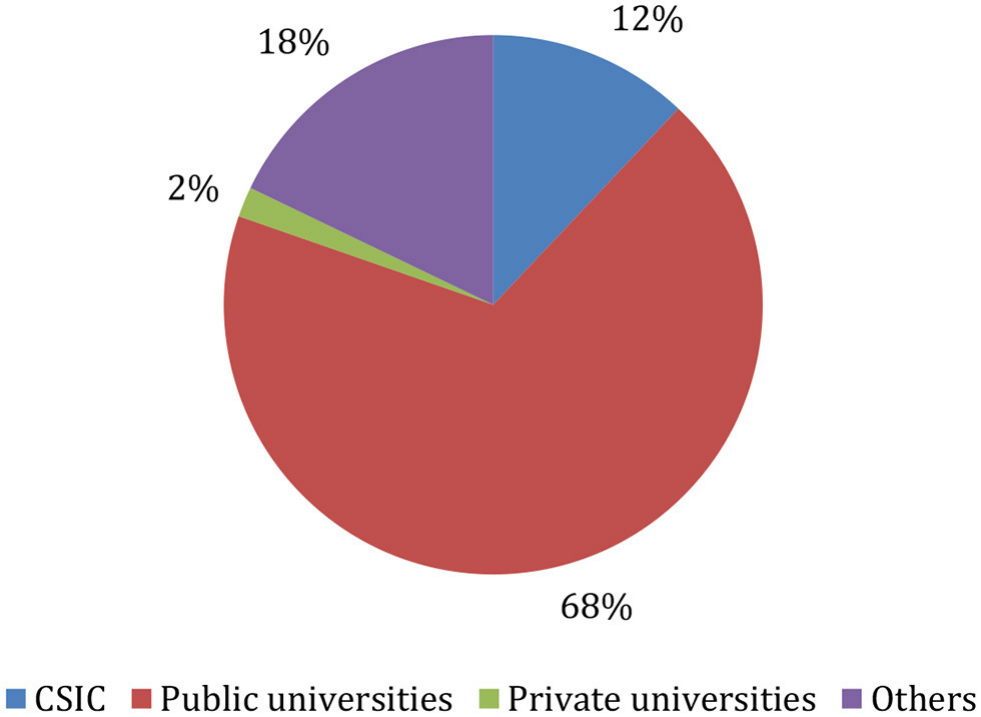
Most cited publications by institution in the period 2005–2014. Percentage. Scopus.

Research in Spain is carried out across a wide range of public and private institutions. There are 75 universities, several large research centers^2^, and more than half a thousand medium and small research centers; besides, around 28,000 companies are registered as innovative firms. In this section, we focus on people's access to research careers in public universities^3^ and the CSIC, the main Spanish public research center. These institutions produce most of the research published in top-tier journals in Spain (Figure 1).

Research results have been gradually, and in an increasingly rigorous manner, incorporated into researchers' recruitment and promotion processes in universities and public research centers. In the 2019–2020 academic year, 120,245 faculty members worked in Spain: 101,305 in public universities and 18,940 in private universities. The main research center, CSIC, employed around 5,000 research personnel.

Faculty careers in Spanish public universities are organized around several categories that can be divided into tenured and non-tenured positions (Escribá-Esteve et al., 2017):

– Tenured positions (in parentheses its name in Spain):
  - Professor or Full Professor with civil servant status (*Catedrático de Universidad*).
  - Associate Professor:
    – With civil servant status (*Profesor Titular de Universidad*).
    – Without civil servant status (*Profesor Contratado Doctor*).
– Non-tenured positions (with non-civil servant status):
  - Assistant Professor/PhD Lecturer (*Profesor Ayudante Doctor*).
  - Teaching Assistant/Non-PhD Lecturer (*Profesor Ayudante*). A PhD is not required.
– Other positions:
  - Part-time Instructor (*Profesor Asociado*).
  - Collaborating Professor (*Profesor Colaborador*).
  - Substitute Professor (*Profesor Sustituto*).
  - Visiting Professor.
  - Emeritus Professor.

Table 1 shows the distribution of academic ranks in public universities in the last academic year for which aggregate data are available. Fifty two percent of the total number of faculty hold tenure-track positions, of which 80% are civil servants. It is important to highlight a large number of part-time faculty (34%). These are practitioners who typically do not engage in research, carry out professional activities outside the university, and only teach a few hours a month.

**Table 1:** Faculty by academic rank.

	**Faculty**	**%**
**Total**	**101,305**	**100.0**
**Tenured positions**	**53,023**	**52.3**
(Full) Professor (CU). Civil servant.	11,791	22.2
Associate Professor (TU + CEU). Civil servant.	27,611	52.1
Associate Professor (*Profesor Contratado Doctor*)	10,682	20.1
Others (TEU). Civil servant.	2,939	5.5
**Non-tenured positions**	**6,279**	**6.2**
PhD Lecturer/Assistant Lecturer (*Profesor Ayudante Doctor*)	5,378	85.7
PhD Lecturer/Assistant Lecturer (*Profesor Lector*)	397	6.3
Teaching assistant (*Ayudante*)	504	8.0
**Other positions**	**42,003**	**41.5**
Visiting professor	952	2.3
Part-time instructor (*Profesor Asociado*)	34,854	83.0
Collaborating Professor (*Profesor Colaborador*)	1,625	3.9
Substitute Professor (*Profesor Sustituto*)	3,560	8.5
Others	252	0.6
Emeritus professor	760	1.8

*Public universities. 2019–2020 academic year*.

*Integrated University Information System (SIIU), University Ministry. CU, Catedrático de Universidad; TU, Titular de Universidad; CUE, Catedrático de Escuela Universitaria; TEU, Titular de Escuela Universitaria. Others refers to positions that have been eliminated by law and there is no new recruitment*.

*The bold values indicate total and subtotals figures*.

Careers at the CSIC are somewhat different. They usually start with a pre-doctoral contract/fellowship, followed by a postdoctoral one, and subsequently, access to the status of researcher. A key element in the assessment of candidates is research, except in the case of pre-doctoral contracts. The postdoctoral stage is a highly competitive career phase, funded by various programs with strong research requirements. Among the most selective programs are the following^4^: *Juan de la Cierva, Torres-Quevedo, Ramón y Cajal, Beatriz Galindo*, ICREA (Catalonia), Ikerbasque (Basque Country), IMDEA (Madrid), and Araid (Aragón). After the postdoctoral stage, the researcher may be offered a permanent contract or be granted the status of career civil servant after passing a competitive examination (*concurso-oposición*).

Table 2 shows the composition of the CSIC research staff according to their position in the research career. The last two rows correspond to scientists with tenured positions at CSIC, namely ^*^Scientific Researcher^*^ and ^*^Research Professor^*^. ^*^Senior Scientists^*^ are postdoc employees that operate under a non-tenured contract. ^*^Senior scientists^*^ may be offered a permanent contract or be granted the status of career civil servant after passing a competitive examination (^*^concurso-oposición^*^). ^*^Scientific Researcher^*^ is a tenured position at CSIC, equivalent to an ^*^Associate Professor^*^ at public universities. Some ^*^Senior Scientists^*^ may get promoted to ^*^Scientific Researchers^*^; however, the competitive examination is also open to external scientists.

**Table 2:** CSIC research staff. 2020.

**Position**	**Researchers**	**%**
Pre-doctoral	1,382	27.2
Postdoctoral	696	13.7
*Ramón y Cajal*	121	2.4
Distinguished researchers	44	0.9
Other researchers	17	0.3
Senior Scientist	1,473	29.0
Scientific researcher	801	15.8
Research Professor	545	10.7
**Total**	**5,079**	**100**

*CSIC internal report*.

**Figure 2:**
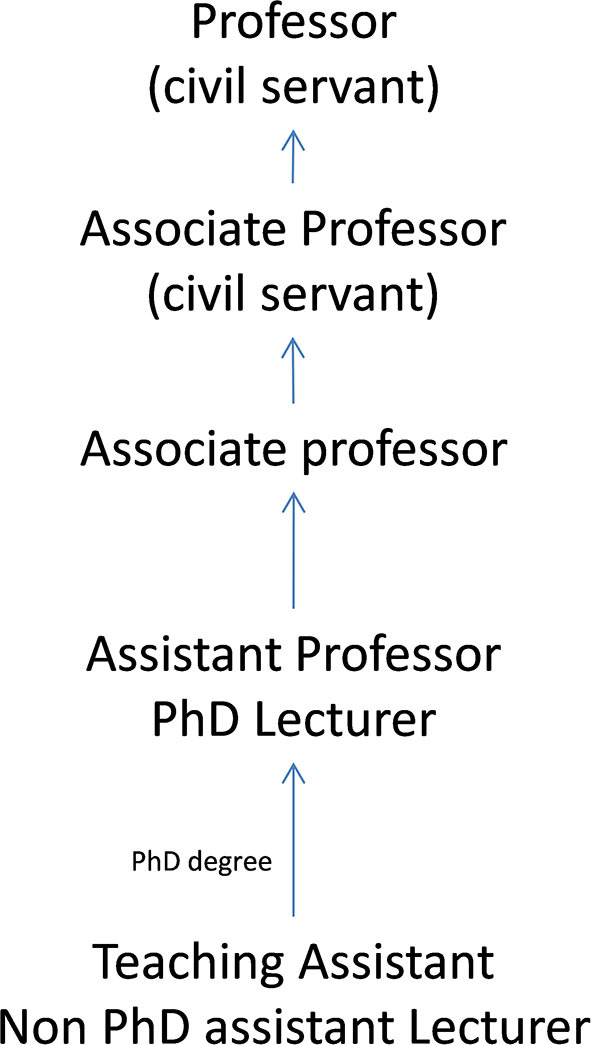
Academic career paths in Spanish public universities.

In most academic promotion processes within universities, accreditation bodies validate and grant the accreditation status that allows access to the competitive selection processes (*concurso-oposición*) held in each university. In Spain, for positions where civil servant status is granted, national accreditation from the National Agency for Quality Assessment and Accreditation (ANECA) is a mandatory process; for other positions and in some autonomous communities, accreditation from the regional agency is required. Figure 2 illustrates the academic career paths in public universities.

By the end of 2001, a system of national qualification, called *habilitación nacional*, was introduced. This was necessary for access to tenured positions. According to the law in force at the time that regulated this system, access to the tenured positions was articulated through a double process. Firstly, there was a national qualification process, and secondly, there was an internal process in which candidates had to pass an examination to be able to work as a university teacher (*concurso-oposición*). Several qualified candidates could compete, although in practice there was no such competition. This system was replaced by another one based on national accreditation, in 2007. Unlike the qualification system in force until then, the offer to a limited number of persons who could qualify was eliminated. Both systems raised the level of merit required from the candidates, especially with regard to research; although they did not solve the problem of researchers' limited mobility, which was one of the objectives of the reform. Even today, 75% of tenured professors in public universities still work at the university where they completed their doctoral thesis, and almost 90% work at a university in their region.

The current accreditation system evaluates different kinds of merits (research, teaching, management, knowledge-transfer activities, and training). Each dimension is assessed in accordance with an alphabetical grading system: A (exceptional), B (good), C (compensable), D (insufficient), and E (special circumstances). According to this system, through which quality of research is assessed, a rating of A in management or in transfer activities makes it possible to compensate accomplishments in research that do not exceed the C level.

In 2015, a reform of the accreditation process raised the level of the merits that had to be achieved. For example, in order to achieve an A grade in research, for a full professor national accreditation in the management field, it is necessary to have published at least 16 articles in first quartile journals of the JCR (Science Citation Index or Social Sciences Citation Index). Once having obtained accreditation, candidates must pass a competitive examination to be able to occupy a position at the university of their choice (*concurso-oposición*), provided this university has offered that position.

In Spain, access to research positions, especially those offered by universities, has been characterized by having to deal with very bureaucratic, closed, and endogamic processes. In order to attract international talent, national (*Ramón y Cajal* and *Juan de la Cierva* grants, among the most important) and regional projects or programs with a great impact on scientific production have been promoted. Ikerbasque and ICREA stand out among the regional ones.

Since 2007, the Basque Foundation for Science, by means of the Ikerbasque annual calls, has been offering permanent research positions to researchers who must pass a very competitive selection process. The positions are automatically granted to candidates who have been awarded any of the European Research Council (ERC) grants. Researches are assigned permanently to Basque universities and research centers. Currently, there are 170 research professors and 42 research associates. Ikerbasque also offers five-year contracts (research fellows) to young researchers with a promising scientific career and international experience. In total, the foundation supports 290 researchers from 36 different countries, publishes about 1,300 indexed papers every year (4,6 papers per researcher), obtaining more than 200,000 citations since its creation 15 years ago, and at present, its researchers are leading 14 ERC grants (Ikerbasque, 2021).

ICREA, for its part, offers tenured, permanent positions to researchers from all over the world, so they come and work in Catalonia. This program, created in 2001, works to integrate ICREA research professors into the Catalan research system. ICREA employs 266 researchers from 27 nationalities who perform their research in 48 different host institutions in Catalonia. In 2019, ICREA published 1,858 papers in the Web of Science (97% in journals), i.e., 7 papers per researcher (ICREA, 2021), with a strong impact. More than a quarter of the ERCs in Spain are led by ICREA researchers.

At the national level, the *Ramón y Cajal* and *Juan de la Cierva* programs stand out. Together, they allow the hiring of 500 to 600 researchers each year. The *Ramón y Cajal* program was launched in 2001, and through its national funding activities, it seeks to attract national and foreign researchers with an outstanding track record in R&D by financing their employment contracts and, also, the creation of permanent jobs for them to be incorporated in research organizations of the Spanish Science, Technology and Innovation System (Ministerio de Ciencia e Innovación, 2021a). Those hired by means of this program mainly carry out their research within universities and research centers. Their productivity is 2.29 articles per year, compared to 0.66 of all other PhDs, as reported in a study by Torres-Salinas and Jiménez-Contreras (2015).

At least 15% of the positions offered by universities to researchers are reserved for those with an accredited PhD who have already completed the *Ramón y Cajal* program and have obtained the *I3 certificate*^5^. When these positions are not filled, other researchers from other programs of excellence (national or international) who have also obtained the I3 certificate may occupy them. This certificate recognizes the quality requirements and standards necessary for proper scientific and technological production and activities that result in an outstanding research career. It was first issued in 2005 in accordance with a law that established the evaluation criteria of the *Incentive Program for the Hiring of Researchers and Intensifying Research Activity* (I3 Program). This program aims to reinforce policies that support the incorporation of PhDs into the Spanish R&D system by offering them permanent positions, whether in universities or other research centers, with the purpose of integrating them into both emerging and consolidated groups. Thus, its objective is to facilitate the recruitment of high-quality Spanish and foreign faculty and researchers, as well as young people with considerable research potential, who wish to join or return to the Spanish science and technology field^6^.

The objective of the *Juan de la Cierva* program is to promote the hiring of people with a doctoral degree by Spanish research organizations or R&D centers for a period of 3 years in order to consolidate the skills acquired at the first stage of postdoctoral training (Ministerio de Ciencia e Innovación, 2021b). This program was designed to complement the *Ramón y Cajal* program, the aim being to define more clearly research careers in Spain, aligning them with the tenure-track positions in other countries, or in the case of researcher profiles, with the *Research Council Academic Fellowships* of the United Kingdom. The *cursus honorum* consists of the following steps (Tapiador, 2006): (1) the best students who wish to follow a research career obtain a training grant or a similar one to do their doctorate; (2) of these, the best qualify for a three-year *Juan de la Cierva* grant at the end of their doctoral thesis; and (3) of these, after completing their postdoctoral training, the best are offered a *Ramón y Cajal* contract, which, following a probationary period, integrates them definitively into a research center.

### Research Bonus

In 1989, a bonus for the research productivity of tenured professors and researchers, known as research sexennial (*sexenio de investigación*), was introduced by law and is currently managed by the ANECA. This incentive has been maintained to date and has had a very positive impact in terms of national research performance. Researchers may apply for it every 6 years with a limit of 6 research periods (36 years); if granted, it is forever. Table 3 shows the salary of university professors and associate professors in Spain and its components: base salary, seniority, a research bonus in the case of having obtained two sexennials, and a teaching bonus—which will be explained later—in case of having obtained three 5-year periods (*quinquenio de docencia*). For each 6-year research period, the best five contributions of the applicant are assessed.

**Table 3:** Tenured professors' gross annual salaries (euros) calculated for 15 years of seniority with all bonuses earned.

	**Salary**	**Seniority**	***Quinquenio***	***Sexenio de***	**Total**
			***de docencia***	***investigación***	
Professor (CU). Civil servant.	44,410	3,093	5,857	3,905	57,264
Associate Professor (TU + CEU). Civil servant.	35,278	3,093	4,744	3,162	46,277
Associate Professor (*Prof. Contratado Doctor*)	32,102	3,251	4,744	3,162	43,259
Others (TEU). Civil servant.	31,101	3,093	4,015	2,676	40,885

*An example from the Complutense University of Madrid*.

*Complutense University of Madrid, based on 2021 data*.

During the first years of its implementation, several changes in the principles and criteria for receiving the research bonus were made in an attempt to reflect the specificities of the different scientific fields (Cabezas-Clavijo and Torres-Salinas, 2015). In 1996, the fields of knowledge and the requirement to publish in journals included in the Journal Citation Reports (JCR) were added to the conditions for the first time, although it was not until 2005 that the criteria for each field were further detailed. In the early years, the percentage of six-year research periods awarded ranged between 60% and 80%, with an increasing trend. Since then, regulations have undergone slight changes, which have in common that requirements have been increased progressively (Sancho and Pérez, 2017).

Table 4 shows the research sexennial statistics for the past eleven academic years. As can be seen from this table, the percentage of civil servants who do not have this bonus is decreasing year after year, although the percentage is still high (18.7%), which means that there are fewer and fewer faculty in the national system with publications that have no international reach, which is the type of research recognized by the sexennial.

**Table 4:** Research sexennial statistics. Civil servants.

	**2009–2010**	**2010–2011**	**2011–2012**	**2012–2013**	**2013–2014**	**2014–2015**	**2015–2016**	**2016–2017**	**2017–2018**	**2018–2019**	**2019–2020**
CS (total)	49,468	49,037	48,423	47,075	47,075	45,839	43,594	42,584	42,199	41,836	41,975
CS with at least one research sexennial	33,116	31,053	N/A	33,360	34,318	34,242	33,210	32,813	32,970	33,322	34,118
Research sexennials (total)	75,133	73,535	N/A	81,605	88,030	91,678	91,847	93,089	95,769	98,549	103,603
Research sexennials (average per CS)	1.5	1.5	N/A	1.7	1.9	2.0	2.1	2.2	2.3	2.4	2.5
CS with at least one research sexennial (%)	66.9	68.25*	69.5*	70.9	72.9	74.7	76.2	77.1	78.1	79.6	81.3
CS with optimal research sexennials (%)	N/A	N/A	N/A	N/A	N/A	N/A	45.4	45.2	45.8	46.4	48.3

*Integrated University Information System (SIIU). CS, civil servants*.

^*^Estimated. N/A, Not available.

An instrument to measure the degree of penetration of this incentive among faculty members is the indicator referred to as the *optimal research sexennial*, which is the number of six-year research periods that a faculty member can achieve over the top theoretical total since he or she obtained a doctorate. Table 4 illustrates the evolution of this indicator. The results suggest an increasing trend, which means that faculty members are doing more and better research. Currently, according to this indicator, Spanish faculty members have been recognized for half of their working lives as having had an impact after they completed their thesis (48.3%).

**Figure 3:**
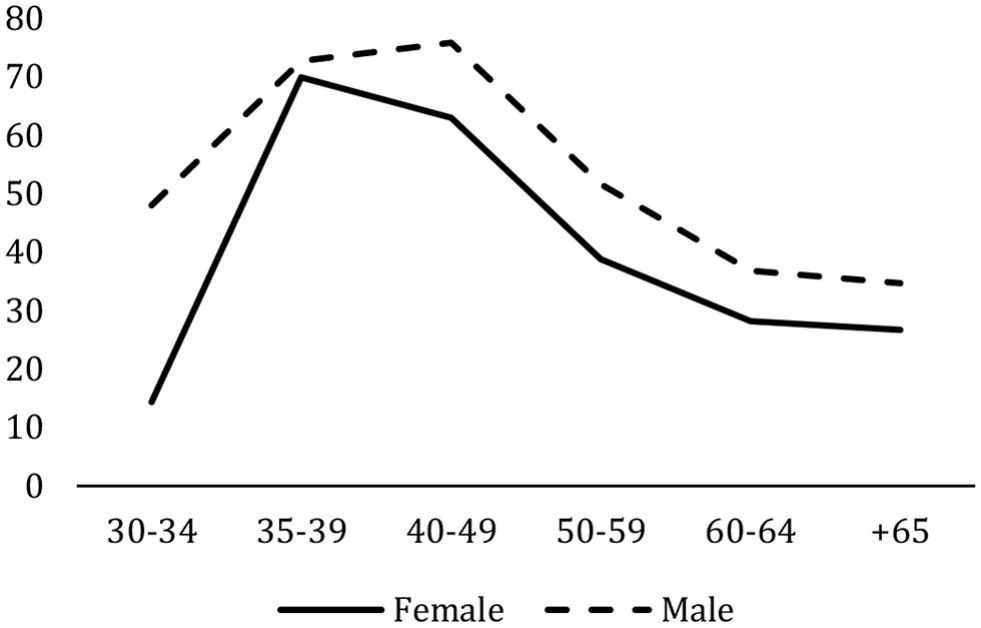
Optimal research sexennials by age and gender. 2019–2020 academic year. Percentage. Integrated University Information System (SIIU).

The optimal research sexennial indicator varies significantly depending on age and gender. As may be observed in Figure 3, civil servants between 35 and 49 years of age are the most productive, with levels of around 70%. This may be explained by the fact that the introduction of the research bonus began to have an impact on the university system and the behavior of researchers in the last years of the 20th century, which coincides with the civil servants who are now around 45 years of age. On the other hand, the large differences by gender are striking. Women obtain similar results than men after the early stages of the career ladder, with a peak at the 35-39 age cohort.

### Research Results

**Figure 4:**
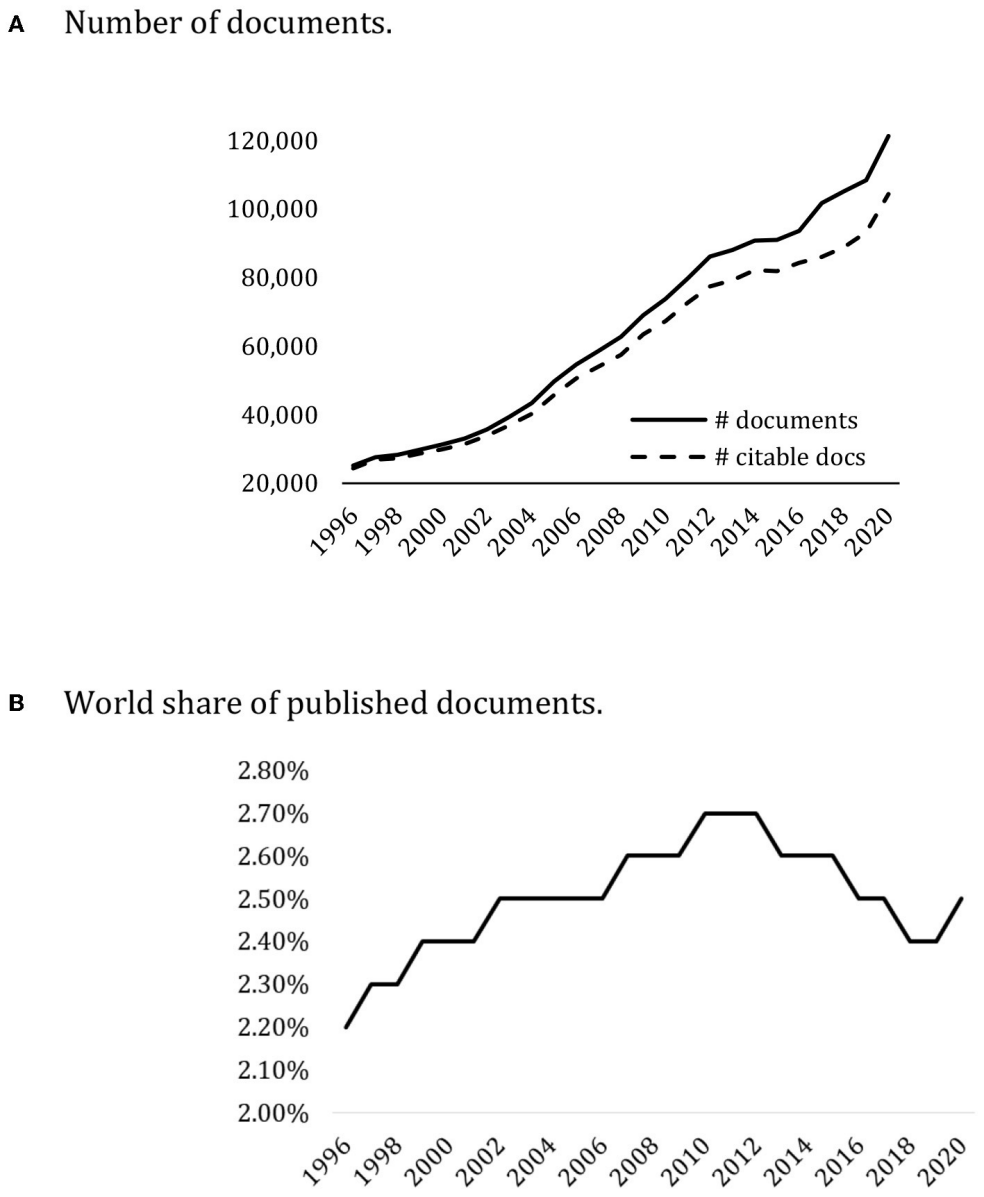
Spanish scientific production. 1996–2020. **(A)** Number of documents. **(B)** World share of published documents. Scopus.

As explained above, over the last thirty years, there have been quite a few changes to the ways in which faculty can access different positions and also in regard to their remuneration through the introduction of the research sexennial pay bonus. This increased rigor for faculty's academic careers together with the new remuneration incentive have helped to greatly increase scientific production in Spain. Since 1996, the number of published documents has multiplied by 4.3 (Figure 4A). It is also worth highlighting its growth in recent years, considering that the number of civil servants in the university system has decreased.

Figure 4B shows the increase in the number of documents published along with Spain's improvement as to its global position in the share of the world's scientific production (2.5% in 2020, ranking 11^th^ in published documents and 10^th^ in citations), even though in recent years, its position has fallen due to the strong growth of countries such as China (which multiplied its production of published documents by 24 in 2020 as compared to 1996), Brazil (x 9.8) or India (x 9).

**Figure 5:**
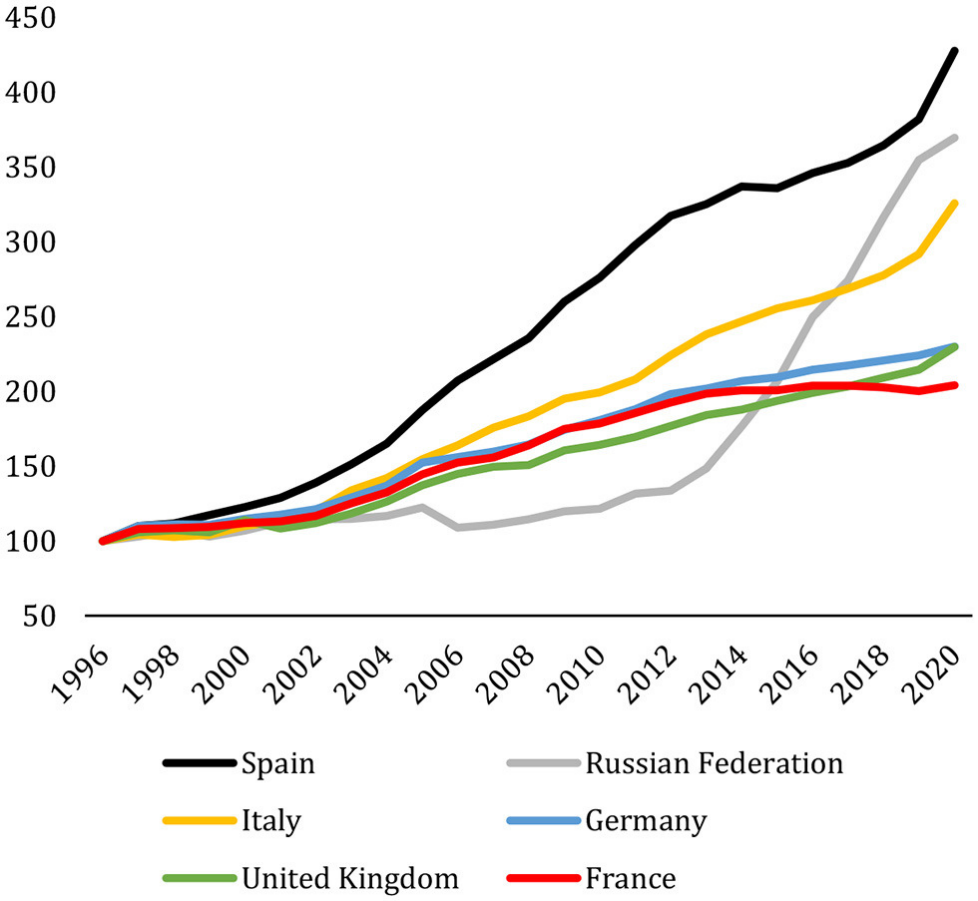
Scientific production (number of documents) of the first European countries in the 2020 world ranking. 1996–2020. 1996 = 100. Scopus.

The excellent performance of these countries hides the also remarkable evolution of Spain when compared with the European countries with the highest share of scientific production in 2020. As illustrated in Figure 5, Spain is the country whose scientific production has grown the most since 1996 with regard to the European countries included in the top 10 of world scientific production.

Two other countries stand up for their performance, as Figure 5 shows, namely Russia and Italy. Recent developments may help in understanding the increase in Russia's scientific production. The establishment in 2014 of the Russian Science Foundation was followed in 2016 by the launching of the Strategy for the Scientific and Technological Development of the Russian Federation^7^ updated by the President in 2018. Universities began offering bonuses to scientists for the publication of papers indexed in international databases; as a consequence, the number of papers showing Russian affiliations more than doubled between 2012 and 2018. The publications surplus consisted mainly of conference papers and articles in the so-called emerging sources. The latter could partly reflect “decisions by international databases to start indexing more local Russian journals” (Schiermeier, 2020). It is, therefore, unclear whether the increase in scientific throughput was solely caused by the production of new publications, although it is quite apparent that the Higher Education landscape is changing fast in Russia, as Figure 5 shows.

For Italy, we refer to the analysis by Giovanni (2015) and the recent study carried out by Bonaccorsi (2020). In his correspondence to The Lancet, Giovanni explains the legislative changes that took place in 2011 regarding competitions for university roles. Giovanni documents how, compared to traditional European competitors such as France, Germany, and the UK, Italy though investing the least “improved the most in terms of scientific production”, to conclude that “with no other way to explain the increase in Italian productivity, I assume that changes in the criteria for access to university careers might have a role”. In the opinion of Bonaccorsi (2020), Italy has carried out two important initiatives than can explain its recent research performance. On the one hand, this country has adopted a regular and mandatory research assessment approach, involving all researchers at universities and at other public research institutions. The 2004–2010 exercise carried out by the Italian National Agency for the Evaluation of Universities and Research Institutes (ANVUR) was one of the largest ever carried out worldwide using peer review in Social Sciences and Humanities, and bibliometrics in Science, Technology, Engineering, and Mathematical disciplines. On the other hand, a major reform of academic recruitment has introduced quantitative indicators as threshold values for candidates to the National Scientific Habilitation based mainly on a massive exercise of rating journals.

In addition to the rise in the number of publications, the percentage of articles and reviews appearing in journals lying in the first quartile of the Web of Science has also steadily increased and now exceeds the 50% of the total number of such types of publications. International collaboration is also on the rise (36% in 2005 to 54.4 in 2019). As reported in the literature (see, for instance, Van Leeuwen, 2009; Moya-Anegón et al., 2013, and Bordons et al., 2015), international collaboration contributes to substantially improving results in terms of quantity and quality of the published research. A discussion around the role of the affiliation of the corresponding author as a measure of leadership has taken place, as reported by Torres-Salinas and Jiménez-Contreras (2015). However, depending on the discipline, positions such as first or last author also constitute signals of leadership and/or seniority (Xiaojun et al., 2010).

**Figure 6:**
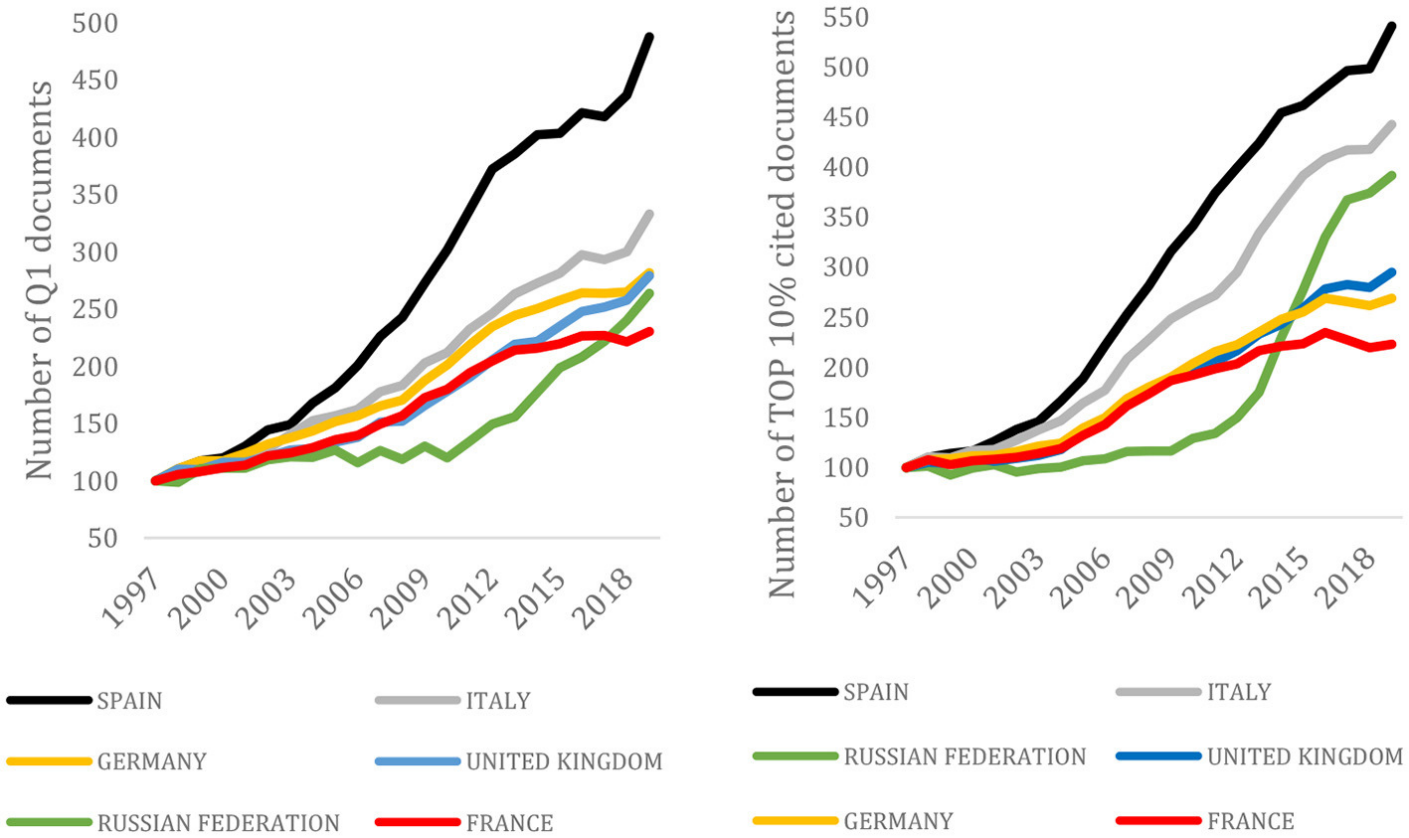
Number of Q1 documents and TOP 10% cited documents of the selected European countries. 1997–2020. 1997 = 100. InCites.

We have used the *InCites* platform from Clarivate Analytics to replicate the results of Figure 5 for publications in the Web of Science (including emerging sources) in the period 2008–2020^8^ in two different scenarios: (1) counting only papers with corresponding author affiliation; and (2) counting papers with corresponding or first or last author. The results obtained are not different from the ones shown in Figure 5; hence, we could safely conclude that the countries shown in the Figure benefited from international collaboration to the same extent over the period 2008–2020. On the other hand, we have also checked the perceived quality of the contributions in the period 1996–2010 through two indicators available from the InCites platform: number of Q1 publications and number of publications in the TOP 10% most cited. Using data gathered from InCites, Figure 6 shows the Q1 ratios from the period 1997–2019. It is apparent that the improvement in the scientific production of papers published with affiliations from Spain extends from the sheer total number of contributions to the more nuanced bibliometric measures of quality (Q1 and TOP 10), as Figure 6 shows.

The improvement in Spain's scientific production can also be seen when examining the global university rankings. Among those measuring research to a greater extent, the most prominent one is the Shanghai ranking (ARWU). Table 5 shows that, in 2020, Spain was the country with more universities among the top 1,000 ARWU universities when normalized by GDP, among those countries accounting for at least 1% of the world's GDP. Data in this table was calculated as follows: for each country, the number of institutions in the TOP X% tier is the world's GDP share of the country times X times 10; the percentage in the column is the ratio of the actual number of universities to the expected number for each tier.

**Table 5:** Number of universities in ARWU 2020.

**Part A**		**Universities in ARWU 1000**				**Part B**		**Universities in ARWU 1000, normalized by GDP**			
		**(sorted by the value of the column “all”)**						**(sorted by the value of the column “all”)**			
**Country**	**% GDP**	**TOP10%**	**TOP25%**	**TOP50%**	**All**	**Country**	**% GDP**	**TOP10%**	**TOP25%**	**TOP50%**	**All**
USA	22.7	41	77	134	206	**ESP**	**1.6**	**0%**	**51%**	**166%**	**255%**
CHN	14.1	6	30	72	144	AUS	1.6	432%	321%	296%	210%
JPN	5.8	3	7	14	40	ITA	2.4	0%	101%	143%	193%
DEU	4.4	4	17	32	49	GBR	3.4	235%	282%	211%	191%
GBR	3.4	8	24	36	65	SCA	1.9	376%	258%	247%	177%
FRA	3.2	5	11	17	30	KOR	1.9	0%	86%	118%	171%
BRA	2.4	0	1	6	22	CAN	2.0	202%	202%	192%	141%
ITA	2.4	0	6	17	46	BLUX	1.7	357%	310%	226%	131%
CAN	2.0	4	10	19	28	DEU	4.4	91%	155%	145%	111%
KOR	1.9	0	4	11	32	CHN	14.1	42%	85%	102%	102%
SCA	1.9	7	12	23	33	FRA	3.2	159%	140%	108%	95%
BLUX	1.7	6	13	19	22	BRA	2.4	0%	17%	50%	92%
AUS	1.6	7	13	24	34	USA	22.7	181%	136%	118%	91%
**ESP**	**1.6**	**0**	**2**	**13**	**40**	JPN	5.8	52%	49%	49%	70%

*Raw data (Part A) and normalized by gross domestic product (Part B)*.

*ARMU (2020) and own elaboration*.

*The bold values indicate the Spanish data*.

**Figure 7:**
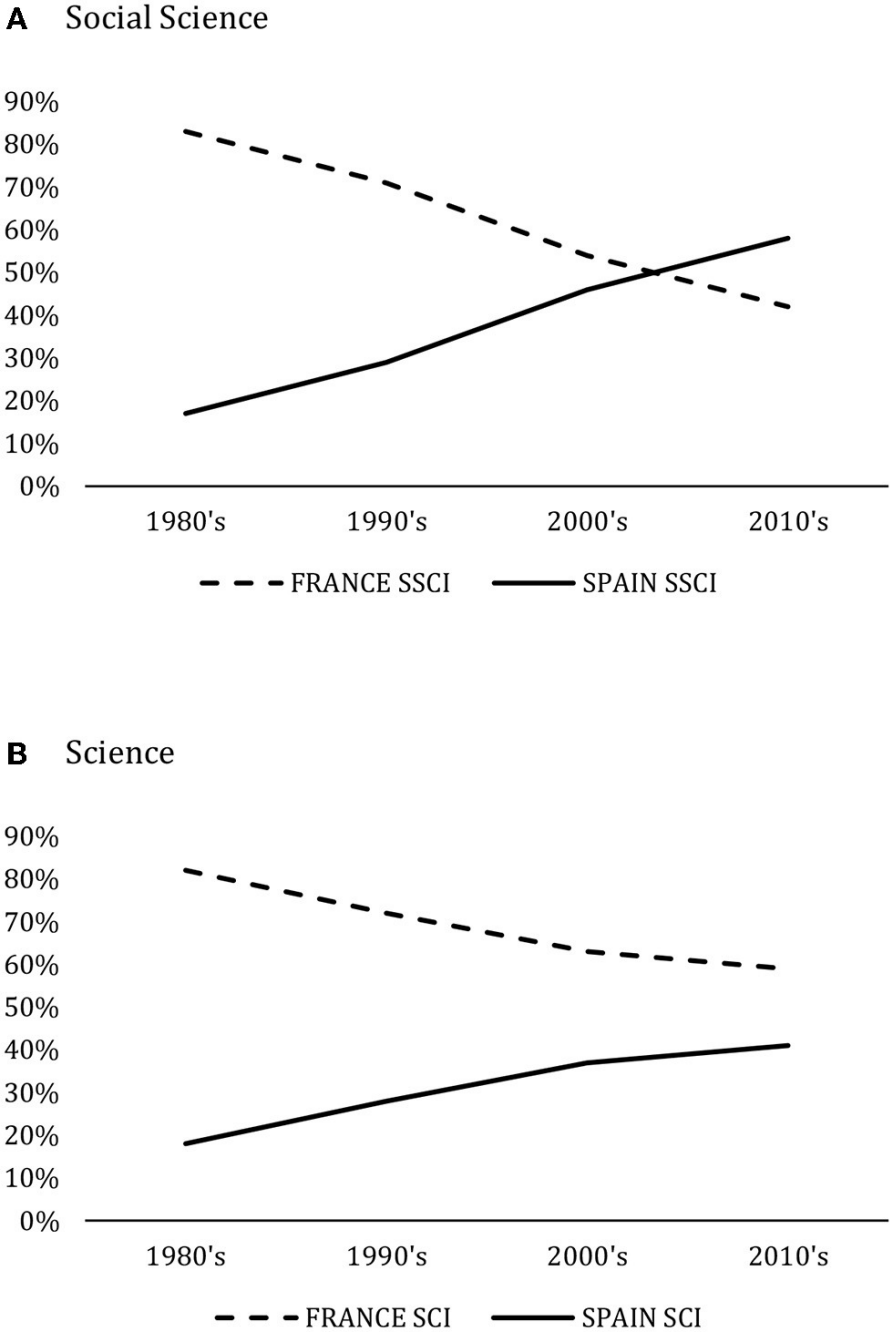
Relative production of Spain versus France. SSCI and SCI. Data taken from the Web of Science core collection corresponding to the period 1980–2019. **(A)** Social Science. **(B)** Science. Own elaboration.

In a recent study, Docampo (2021) highlighted the positive impact of introducing both a research bonus (in the 1990s) and a national accreditation system on scientific production in Spain and compared it to other European countries. Figure 7 shows that the evolution of the relative production of Spain with respect to France in Science and Social Science along the past 40 years has been very positive, even surpassing France's share in Social Science despite the differences in population and wealth between the two countries. The data represented in the figures correspond to the relative share of the aggregated scientific production of both countries over four decades.

## Teaching

Teaching in Spain is assessed by considering two main parameters of a faculty's academic careers. First, faculty members' teaching achievements are assessed when they apply for different university positions (Figure 2), although teaching merits are given less weight and are evaluated in a less objective way than research merits, merely considering the number of years of teaching experience and variety in teaching (essentially the different levels: bachelor's, master's and doctorate). The second parameter is a pay bonus for teaching quality, called *quinquenio de docencia* (“5-year teaching period”), which was created by law in 1989 with the aim to improve university teaching. It is granted every 5 years, with a limit of six bonuses per person (30 years in total).

Since its implementation, each university has evaluated its faculty and, as a general rule, all faculty members receive this bonus. So it really has become a seniority bonus, rather than a teaching quality bonus. Table 6 shows the success rate in the application for this bonus at the University of Valencia and at the University of Vigo. As can be seen, all civil servant teachers apply for this bonus every 5 years (applications are made by, more or less, one fifth of faculty teachers), and all faculty teachers (almost 100%) obtain it, which in practice means it is seniority pay. For this reason, the national accreditation agency (ANECA) is now developing a new bonus, called *sexenio de docencia* (6-year teaching period), to be assessed outside the governance of universities.

**Table 6:** Success rate of the *quinquenios* at the University of Valencia and the University of Vigo.

	**Faculty^a^**	**Applications^b^**	**Accepted^b^**	**%**
University of Valencia	1,933	408	405	99.3
University of Vigo	735	169	168	99.4

a *Number of civil servants. Academic year 2019–2020*.

b *Year 2019 for the U. of Vigo and 2020 for the U. of Valencia*.

*University of Valencia and the University of Vigo*.

The results of teaching in Spain over the last few years have improved, mainly because of the university reform carried out under the influence of the Bologna Process. Spain enacted a law in order to adapt its university system to the new European framework in 2007. The new university degrees (bachelor's, master's, and doctoral degrees) were streamlined, and teaching methodologies were improved. In the 2008–2009 academic year, the first degrees adapted to the European Higher Education Area were offered. In 2007, still without the effect of the new degrees, the percentage of credits passed out of the total number of credits enrolled was 63.8% in public universities. In recent years, this percentage has increased by more than 20 points.

**Figure 8:**
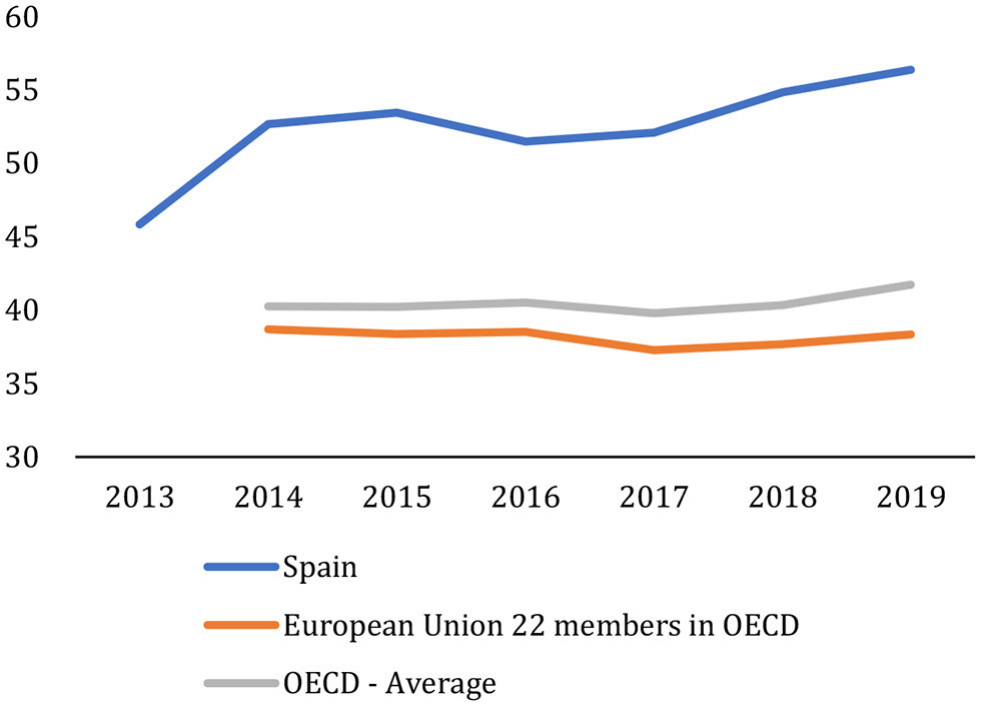
First-time tertiary graduation rates for students below the age of 30. Graduation rates in 2018. Percentage. OECD (2021) and own elaboration.

In general, the teaching results obtained on the basis of the success rates and evaluation indicators are good and comparable to those of other OECD or EU countries, but not in terms of employment. Graduation rates offer a signal of whether government and universities initiatives have successfully increased the share of people who graduate from tertiary education. The differences in graduation rates among countries reflect the performance of the national higher education systems, the variety of systems and programs available, and other country-specific factors, such as current social norms and economic performance (OECD, 2021). The first-time tertiary graduation rate^9^ for students below the age of 30 in Spain (Figure 8) is now 56%, well above the average for OECD countries (42%) and EU22 countries (38%). Since 2013, Spain has improved this indicator by 10 points, widening the difference with the OECD and EU countries.

**Figure 9:**
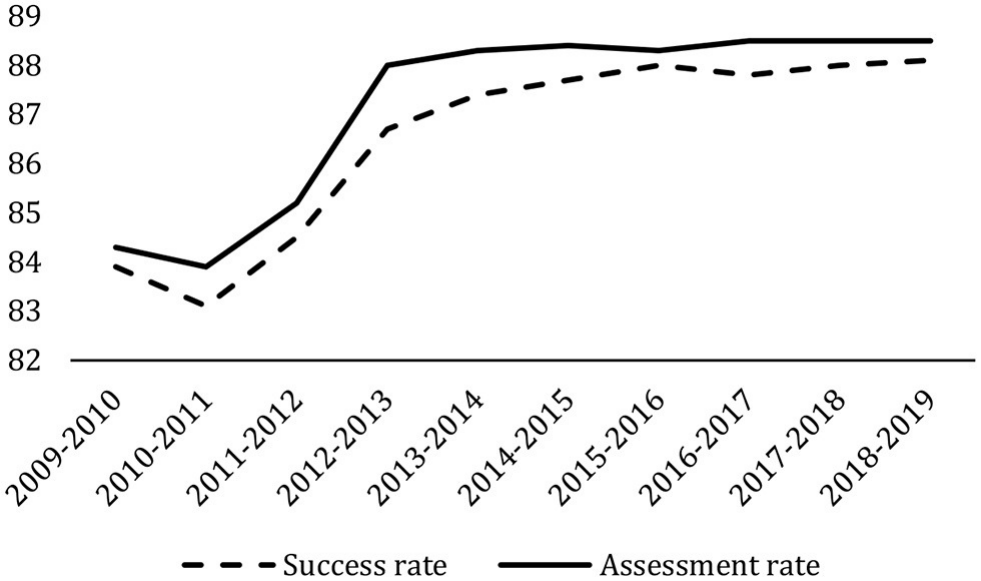
University success and assessment rates in Spain. Percentages. Academic years 2009/2010–2018/2019. Integrated University Information System (SIIU).

The success rate, measured as the number of credits earned (excluding those “credits taken by students who did not do the exam”), was 88.1% in the academic year 2018–2019 (latest data available) with there being a clear positive trend since 2009–2010, which stabilized from the academic year 2015–2016 onwards (Figure 9). The assessment rate measures the number of credits evaluated over the number of credits enrolled as a percentage. This indicator shows a similar evolution to the success rate and has remained above 88% beyond 2012–2013. These indicators have improved mainly due to the adaptation of the Spanish university degrees to the EHEA requirements. No noticeable effect was observed after introducing the 5-year teaching bonus in 1989 (*quinquenio*), nor with the national accreditation processes in 2001.

The results of university students' transitions into the labor market are much less satisfactory than their academic outcomes. However, university graduates have experienced a positive evolution of their unemployment rate in comparison with that of the total active population in Spain. In 2020, the unemployment rate among university graduates was 9.2%, and that of the total Spanish population was 15.5%.

With such a high unemployment rate it is clear that the ability of Spain's educational system to get its university graduates into the labor market is not good, although a large part of the responsibility lies with Spain's endemic employment problem, with one of the highest unemployment rates in the EU. Table 7 shows the percentage of university graduates that were hired in the 4 years following completion of their studies in 2009–2010 and 2013–2014. There is an improvement in the data between 2010 and 2013. However, during the first year, less than half of the graduates found jobs. This proportion rises to just over 70% after 4 years.

**Table 7:** University graduates' employment rate in the 4 years after graduation.

	**+1 year**	**+2 years**	**+3 years**	**+4 years**
Graduates in 2009/2010	43.4	55.6	58.6	64.4
Graduates in 2013/2014	47.0	59.6	67.8	72.3

*Integrated University Information System (SIIU). Ministry of Universities and Vocational Education*.

**Figure 10:**
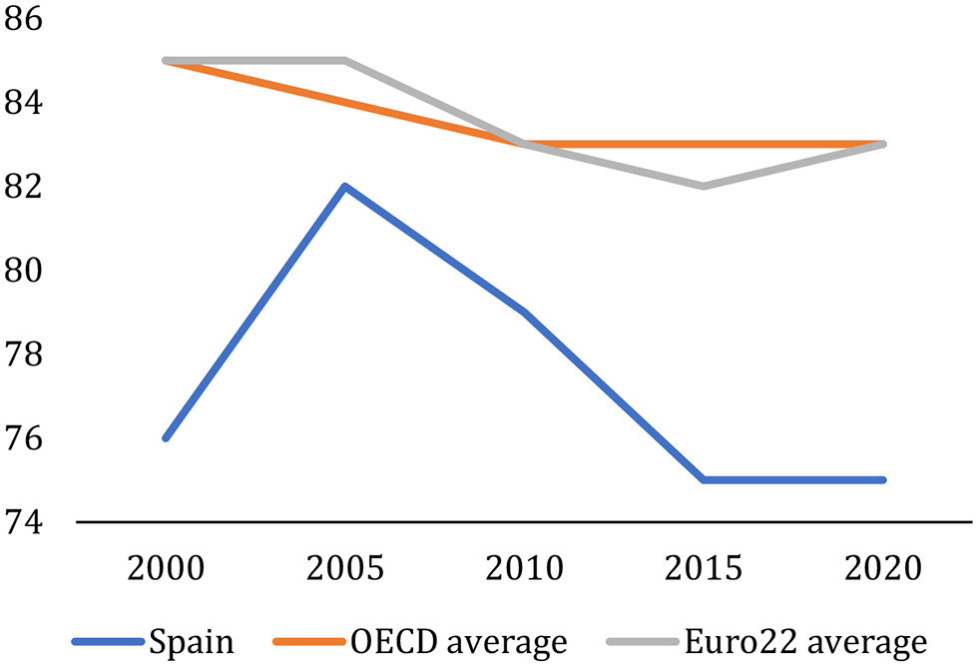
Trends in employment rates. Tertiary education. Percentage of 25–34 year-olds employed in this age bracket. 2000, 2005, 2010, 2015, and 2020. OECD indicators, Education at a Glance (OECD, 2017, 2021).

This data is not good when compared with other groups of reference countries (Figure 10). In the last 20 years, the employment rate among graduates aged 25–34 in Spain has worsened and the gap with respect to the OECD countries and the EU22 group countries has widened.

Along with very low levels of employability during the first year is the lack of fit between what graduates study and the work they carry out. As Table 8 shows, only half of the students are employed in jobs that correspond to their level of studies, and after 4 years, 40% still do not have suitable jobs. In addition to the fact that the data has not improved between 2010 graduates and 2014 graduates, especially during the first year after graduation.

**Table 8:** Rate of graduates employed in their professional category in the following 4 years to graduation.

	**+1 year**	**+2 years**	**+3 years**	**+4 years**
Graduates in 2009/2010	48.5	53.5	53.8	55.5
Graduates in 2013/2014	48.2	54.0	57.7	60.7

*Integrated University Information System (SIIU). Ministry of Universities and Vocational Education*.

## Knowledge Transfer

Several countries use a broader set of indicators to assess knowledge transfer activities (e.g., see Zacharewicz et al., 2019 and Luwel, 2021). Assessing the third mission of the university is a very challenging task since it involves taking into account many and heterogeneous activities undertaken by faculty and researchers. For every teaching and research position, merits that can also be classified as transfer merits are valued, and many of them are sometimes confused with research merits.

In Spain, the evaluation of knowledge transfer, and innovation activities undertaken by university faculty and researchers of public research organizations began to be regulated at the end of 2018. It was launched as a pilot project, with a new salary component called *sexenio de transferencia* (6-year transfer period). This new payment led to a complementary process to the one that takes place for the *research sexennial* (6-year research period). Together with the *quinquenio de docencia* (5-year teaching period), assessment of universities' triple mission was completed: research, teaching, and knowledge transfer.

The call is considered experimental and pioneering at the global level as no other country has undertaken the task of attempting to measure and assess the activities of the third mission in such a broad sense (Rodríguez-Conde, 2020). The objective of this new incentive is to recognize the impact, outreach, and visibility of the knowledge transfer activities carried out. The evaluation process for the 6-year transfer period is similar to that of the research sexennial: the evaluation is carried out on five contributions presented by the applicant for a period of 6 years, although candidates must have at least one 6-year research period granted in order to be able to apply for it. Four types of merit are considered: transfer through the training of people, transfer through activities with institutions, transfer generating economic wealth, and transfer creating social value (see Supplementary Material).

To date, there has been only one call for applications: the 2018 pilot project. In total, 16,791 applications were submitted, where 31.55% were from the engineering and architecture fields, 29.33% from social and legal sciences, 21.25% from the field of science, 9.82% from health sciences, and 8.04% from arts and humanities.

The university community has described the results as very disappointing, given that only 54.8% of the requested bonuses have been granted. The results are very heterogeneous in terms of gender and the evaluated fields. While 65.5% of men obtained a favorable evaluation, only 34.5% of women obtained this result. The difference was especially notable in fields from 6 to 9. For instance, in field 7, 87% of the men were successful, whereas the percentage of women that received the bonus was only 13%. Early studies have observed a clear gender bias in the evaluation of this pilot project, showing that more weight has been given to the transfer that generates economic value, as opposed to other types of transfer in which more women participate (López-Díaz and Pereira-Gómez, 2021). These findings produced a strong reaction among the universities, resulting in a substantial revision of this new incentive, for which its second call has not yet launched.

It is still too early to evaluate the impact of this new productivity bonus on the transfer activities of universities and research centers. Hence, we will have to wait a few years to see how this bonus, which should carry greater recognition of the transfer of knowledge of social value, often more informal and without objectively measured economic value, is consolidated and what will be the extent of its impact.

## Discussion and Conclusions

In Spain, faculty members' and researchers' activities are assessed for three main reasons: applying to university teacher and researcher positions, developing their academic careers, and calculating their individual remunerations. This assessment covers three dimensions of university activity: teaching, research, and knowledge transfer. Over the last 20 years, the level of career-related requirements has been raised considerably with the introduction of a habilitation system in 2001, which was followed by a new accreditation system in 2007, both national in scope and beyond the control of individual universities.

On the other hand, during the last three decades, three distinct financial incentives have been introduced for tenure-track positions: one for teaching achievements, which was introduced in 1989, is awarded every 5 years, and is managed by the universities with a limit of 6 bonuses (30 years); a research incentive that was created in 1989, is available every 6 years, and is managed outside the universities with a limit of 6 bonuses (36 years); and another one that has been recently created to reward knowledge-transfer achievements. The latter is also administered by external agencies and has a limit of 6 bonuses (36 years, but these are added to those of the research sexennial). These measures have reached very different outcomes. The teaching bonus is evaluated by the universities and the results are positive almost to a 100%; therefore, it is clear that this incentive does not play any role whatsoever. The research bonus, which is awarded after being evaluated by an external agency, has been the most remarkable individual incentive for faculty and researchers in Spain for the past 30 years. The introduction of this incentive scheme was not an immediate success, since it had to overcome all kinds of corporatist pressures. However, after the first 5 years, it earned the respect of the research community because it was accepted as a transparent and reliable procedure for evaluating individual research performance across all disciplines. Furthermore, it has contributed significantly to professionalizing research activities in Spain and is arguably the main reason behind the growth in the scientific production of Spanish academic institutions and research centers.

Although the accreditation system and public contests for academic positions have been straightened up during the past few years, they still do not help boost faculty's mobility within the academic system. Other programs targeting talent attraction and retention, such as Ramón y Cajal and Juan de la Cierva, have successfully contributed to the mobility of researchers across the Spanish higher education system. Special programs such as Icrea and Ikerbasque have been particularly successful in attracting international talent to Spanish institutions.

Teaching results depend largely on a country's higher education system and other social and economic variables, such as its unemployment rate. On the one hand, our findings for Spain show an improvement in the pass and graduation rates over recent years, more due to the implementation of the Bologna process than to the introduction of a national evaluation system (habilitation/accreditation) in the first years of this century, or the introduction of bonuses for improving teaching productivity in 1989. On the other hand, the outcomes in terms of employability and job suitability are poor. Moreover, there has been no convergence with other benchmark countries in the last 15 years. This result is a consequence of the high Spanish unemployment rate and also of the system's inability to prepare graduates for their transition into the labor market.

In Spain, knowledge-transfer achievements have begun to be measured with a new bonus (transfer sexennial). However, it is still too early to tell whether this will change the behaviors of faculty and researchers. Of the three missions carried out by the faculty and research staff on behalf of their university, knowledge transfer is presently perhaps the most difficult to measure, given the lack of widely accepted indicators for all disciplines and for those activities that cannot be monetarily quantified. This incentive is, therefore, currently under review. And there is an additional problem since the limit of the transfer bonus is added to the research bonus limit, which is set at 6. Hence, if a researcher obtains as many bonuses as possible in 18 years (i.e., 2 bonuses every 6 years), he or she will cease to have incentives in their salary thereafter.

Table 9 summarizes the instruments which have had the greatest impact on faculty performance at the individual level, both in respect of faculties' academic careers and the calculation of the additional compensation for excellence in teaching, research, and knowledge transfer, and according to whether the assessment is made within or outside the university. In our opinion, bearing in mind these results, the most positive impact achieved is with those incentive schemes that were taken out of the academic control of university governance. Consequently, this finding should be taken into account when undertaking future initiatives and reforms.

**Table 9:** Impact of the university career and salary bonuses.

		**Locus of control**		**Impact**
		**University**	**Outside the university**	
**Tenured positions (professor and associate professor)**	Accreditation (necessary condition)		x	+
	Recruitment	x		-
**Bonus**	Research sexennial		x	+
	Five-year teaching period	x		-
	Six-year transfer period		x	?

*Own elaboration*.

## Data Availability Statement

The raw data supporting the conclusions of this article will be made available by the authors, without undue reservation.

## Author Contributions

CA gathered the information for figures and tables and contributed to the preparation of the manuscript. VS and DD were equally responsible for the conception of the paper and for the writing of the manuscript. All authors contributed to the article and approved the submitted version.

## Funding

The work of DD has received financial support from the Xunta de Galicia (Centro Singular de Investigación de Galicia Accreditation 2019-2022) and the European Union (European Regional Development Fund—ERDF). The work of VS has received financial support from the Generalitat Valenciana (AICO/2021/309).

## Conflict of Interest

The authors declare that the research was conducted in the absence of any commercial or financial relationships that could be construed as a potential conflict of interest.

## Publisher's Note

All claims expressed in this article are solely those of the authors and do not necessarily represent those of their affiliated organizations, or those of the publisher, the editors and the reviewers. Any product that may be evaluated in this article, or claim that may be made by its manufacturer, is not guaranteed or endorsed by the publisher.

## Footnotes

^1^See the universidades.gob.es website.

^2^Among the largest centers are the following: Consejo Superior de Investigaciones Científicas (CSIC), Centro de Investigaciones Energéticas, Medioambientales y Tecnológicas (CIEMAT), Instituto Geológico y Minero de España (IGME), Instituto Español de Oceanografía (IEO), Instituto Nacional de Investigación y Tecnología Agraria y Alimentaria (INIA), Instituto de Astrofísica de Canarias (IAC), and Instituto de Salud Carlos III (ISCIII).

^3^Most of the faculty members in universities are teaching and research staff, although at the individual level, very different situations arise: some are essentially only researchers, while others are basically only teachers, but most have more balanced mixes of teaching and research.

^4^Researchers entering university careers also have access to these programs.

^5^Ministry of Universities' call for I3 certifications for teaching and research staff in the Spanish university system.

^6^Obtained from Spanish legislation: ORDEN ECI/1520/2005 and BOE 296, 10-11-2020, pp: 98612-98622.

^7^See http://kremlin.ru/acts/bank/41449 (in Russian) or http://online.mai.ru/StrategySTD%20RF.pdf (unofficial translation).

^8^Results concerning affiliation positions are available from *InCites* only after 2008.

^9^“First-time tertiary graduates refer to students who graduate for the first time with a tertiary degree, regardless of the education program in which they are enrolled” (OECD, 2021: 205).
