# Supplementary material for: Research, Teaching, and Knowledge Transfer Assessment in Spain: Strategies and Results
Source: Front Res Metr Anal. 2022 Jun 29;7:817031. doi: 10.3389/frma.2022.817031 (PMC9277222; doi:10.3389/frma.2022.817031)
Supplement: Supplementary file 1 [file Data_Sheet_1.docx]

**Supplementary Material**

**Table A. Type of contributions and indicators in the six-year transfer period (2018 edition)**

| Type of contributions | Indicators |
| --- | --- |
| 1. Transfer through the training of researchers | 1.a. The number of people hired under R&D projects and contracts during the period assessed.  1.b. Industrial theses (companies or relevant non-teaching institutions)  1.c. Number of people hired in entrepreneurship activities |
| 2. Transfer of proprietary knowledge through activities in other institutions | 2.a. Periods in other institutions.  2.b. Memberships in committees of high relevance in the field. |
| 3. Transfer that generates economic wealth | 3.a. Royalty income.  3.b. Participation in contracts and transfer projects.  3.c. Partner in spin-offs during the period.  3.d. Number of patents, utility models, trademarks, etc. |
| 4. Transfer creating social value | 4.a. Participation in agreements or contracts with non-profit entities or public administrations and projects for social development awarded in competitive calls for proposals.  4.b. Publications (books, book chapters, or articles) (exceptionally, exhibitions and materials), dissemination activities and dissemination of research in audiovisual media, professional dissemination, etc. |

Source: ANECA.

**Table B. Frequency of merits accomplished by field of knowledge**

| Indicator | Frequency |
| --- | --- |
| 1.a. The number of people hired under R&D projects and contracts during the period assessed. | Widely used in technological fields |
| 1.b. Industrial theses (companies or relevant non-teaching institutions) | This merit very little used, demonstrating the poor relationship between universities and industry in Spain compared to other European countries, in which this relationship is stronger. |
| 1.c. Number of people hired in entrepreneurship activities | In general, this indicator is scarcely used.  It has a greater use in the scientific and technological fields than in the humanities and socio-legal fields. |
| 3.a. Royalty income. | It is little used in general, except in fields of pharmacy or engineering. |
| 3.c. Partner in spin-offs during the period. | Frequently used in the scientific and technological fields and very sporadically in the legal, social, and humanities fields. |
| 3.d. Number of patents, utility models, trademarks, etc. | Hardly used in the humanistic and legal fields. |

Note: This Table provides information about the frequency with which merits are used by type of indicator and field of knowledge (only the merits that have produced the greatest differences are shown).

Source: Rodríguez-Conde (2020).

**Table C. Six-year transfer period. 2018 pilot call**

**(overall results presented in November 2020).**

| Field | Applications | Overall success | Men’s success | Women’s success |
| --- | --- | --- | --- | --- |
| 1. Chemistry | 888 | 52.4% | 61.0% | 39.0% |
| 1. Physics and Mathematics | 1,193 | 62.2% | 74.7% | 25.3% |
| 1. Natural Sciences and Biochemistry | 1,487 | 54.2% | 64.4% | 35.6% |
| 1. Health Sciences | 1,646 | 51.3% | 58.0% | 42.0% |
| 1. Chemical and Materials Engineering | 1530 | 52.3% | 60.7% | 39.3% |
| 1. Mechanical and Navigational Engineering | 798 | 70.1% | 81.7% | 18.3% |
| 1. Electronics and Systems Engineering | 1,514 | 77.4% | 87.0% | 13.0% |
| 1. Computer Engineering | 1,016 | 71.0% | 82.4% | 17.6% |
| 1. Architecture and Civil Engineering | 448 | 66.3% | 78.6% | 21.4% |
| 1. Economic Sciences | 855 | 53.7% | 63.5% | 36.5% |
| 1. Business Sciences | 930 | 51.4% | 58.3% | 41.7% |
| 1. Social and Behavioral Sciences | 1,218 | 50.1% | 52.1% | 47.9% |
| 1. Arts and Humanities | 1,367 | 50.9% | 56.6% | 43.4% |
| 1. Legal Sciences | 1,243 | 50.3% | 53.9% | 46.1% |
| 1. Educational Sciences | 658 | 52.1% | 60.2% | 39.8% |
| **Total** | **16,791** | **54.8%** | **65.5%** | **34.5%** |

Source: ANECA.
